# Multi-contrast late enhancement CMR determined gray zone and papillary muscle involvement predict appropriate ICD therapy in patients with ischemic heart disease

**DOI:** 10.1186/1532-429X-15-57

**Published:** 2013-06-26

**Authors:** Yuesong Yang, Kim A Connelly, Tawfiq Zeidan-Shwiri, Yingli Lu, Gideon Paul, Idan Roifman, Mohammad I Zia, John J Graham, Alexander J Dick, Eugene Crystal, Graham A Wright

**Affiliations:** 1 Imaging Research and Schulich Heart Center, Sunnybrook Health Sciences Centre, University of Toronto, 2075 Bayview Avenue, Toronto, Ontario, Canada; 2 Division of Cardiology and Keenan Research Centre in the Li Ka Shing Knowledge Institute, St. Michael’s Hospital, 30 Bond Street, Toronto, Ontario, Canada; 3 Ottawa Heart Institute, 42 Ruskin Street, Ottawa, Ontario, Canada

**Keywords:** Infarct heterogeneity, Cardiovascular magnetic resonance, Late gadolinium enhancement, Ischemic heart disease, Implantable cardioverter-defibrillator

## Abstract

**Background:**

Myocardial infarct heterogeneity indices including peri-infarct gray zone are predictors for spontaneous ventricular arrhythmias events after ICD implantation in patients with ischemic heart disease. In this study we hypothesize that the extent of peri-infarct gray zone and papillary muscle infarct scores determined by a new multi-contrast late enhancement (MCLE) method may predict appropriate ICD therapy in patients with ischemic heart disease.

**Methods:**

The cardiovascular magnetic resonance (CMR) protocol included LV functional parameter assessment and late gadolinium enhancement (LGE) CMR using the conventional method and MCLE post-contrast. The proportion of peri-infarct gray zone, core infarct, total infarct relative to LV myocardium mass, papillary muscle infarct scores, and LV functional parameters were statistically compared between groups with and without appropriate ICD therapy during follow-up.

**Results:**

Twenty-five patients with prior myocardial infarct for planned ICD implantation (age 64±10 yrs, 88% men, average LVEF 26.2±10.4%) were enrolled. All patients completed the CMR protocol and 6–46 months follow-up at the ICD clinic. Twelve patients had at least one appropriate ICD therapy for ventricular arrhythmias at follow-up. Only the proportion of gray zone measured with MCLE and papillary muscle infarct scores demonstrated a statistically significant difference (P < 0.05) between patients with and without appropriate ICD therapy for ventricular arrhythmias; other CMR derived parameters such as LVEF, core infarct and total infarct did not show a statistically significant difference between these two groups.

**Conclusions:**

Peri-infarct gray zone measurement using MCLE, compared to using conventional LGE-CMR, might be more sensitive in predicting appropriate ICD therapy for ventricular arrhythmia events. Papillary muscle infarct scores might have a specific role for predicting appropriate ICD therapy although the exact mechanism needs further investigation.

## Background

Sudden cardiac death is a major cause of mortality in patients with ischemic heart disease (IHD). This mortality is partially attributable to malignant ventricular arrhythmias (VA) after myocardial infarction (MI). These malignant arrhythmias can be terminated by implantable cardioverter-defibrillator (ICD) therapy. Currently, poor left ventricular ejection fraction (LVEF) is the primary index that is used to decide whether or not to implant an ICD [1-6]. However, many patients with poor LVEF may not benefit from the ICD implantation as the annual firing rates are approximately 5% [1,5]. Thus a better risk stratification strategy may help improve the efficacy of ICD therapy.

Late gadolinium enhancement (LGE) cardiovascular magnetic resonance (CMR) can identify infarct heterogeneity which may represent a critical substrate for arrhythmogenesis in patients post MI [7-9]. Infarct heterogeneity indices including myocardial scar tissue and peri-infarct gray zone (GZ) may have the potential to correctly predict VA events after ICD implantation in patients with ischemic or non-ischemic cardiomyopathy [10-12]. These indices are determined by signal intensity thresholds or the full-width half-maximum (FWHM) method using conventional LGE-CMR [13-15]. Recently a multi-contrast late enhancement (MCLE) technique was developed to yield improved visualization of infarct tissue including a better identification of papillary muscle (PM) involvement with a potential of accurate quantification of infarct heterogeneity [16-18]. Automated segmentation and classification of infarct heterogeneity can be realized using MCLE and thus a better reproducibility might be achieved [19]. While PM involvement is a recognized source of VA events in patients with IHD [20,21], the specific role of PM-MI in the prediction of appropriate ICD therapy has not been established yet. In this study, we hypothesize that the extent of peri-infarct GZ and PM-MI scores determined by MCLE may have the potential to more accurately predict appropriate ICD therapy in patients with IHD post ICD implantation.

## Methods

### Patient population

The institutional research ethics board at Sunnybrook approved the study protocol and informed consent was obtained in all subjects. In this study twenty-five patients with prior myocardial infarction referred to our institution for ICD implantation for primary or secondary prevention were enrolled for a pre-implantation CMR study. Patients with MR-incompatible implants such as pacemakers, intra-cranial aneurysmal clips and other contraindications for CMR were excluded from study. After the CMR examination and ICD implantation, the patients were followed up in an ICD clinic on a quarterly basis.

### CMR protocol and image analysis

The CMR protocol included LV functional parameter assessment using steady-state free precession (SSFP), as well as LGE-CMR using an inversion recovery fast gradient echo (IR-FGRE) and MCLE post double-dose Gd injection [17-19]. All CMR studies were performed on a 1.5 T GE Signa HDx system (GE Healthcare, Milwaukee, USA) with ECG gating and using an eight-channel phased-array cardiac coil. First, a short-axis oblique SSFP stack of slices covering the whole left ventricle based on a 3-plane localizer sequence were acquired for LV function assessment. Typical SSFP CMR parameters were as follows: bandwidth (rBW) ±125 kHz, flip angle 45°, views per segment (VPS) 16, TR/TE 3.7/1.6 ms, field of view (FOV) = 32 cm, image matrix = 256×192, and slice thickness 8 mm. 20 phase-resolved images over the whole cardiac cycle were acquired in a breath hold. Post-contrast LGE-CMR images using IR-FGRE which was usually performed first and followed by MCLE were acquired 10–20 minutes after a double-dose intravenous bolus injection of Gd-DTPA (Magnevist®, Bayer Inc., Canada; Equivalent to 0.2 mmol/kg) using the same short-axis oblique localization as the SSFP LV function study. Depending on the null point of normal myocardium, the inversion time (TI) varied from 200 to 300 ms in IR-FGRE. Typical IR-FGRE CMR parameters consisted of the following: TR/TE 6.0/3.0 ms, rBW ±31.5 kHz, flip angle 20°, VPS 20, number of excitations (NEX) 2. The delay time (TDEL) was chosen to yield images in mid- to late diastolic phase. Approximately 20 heartbeats (18-second breath-holds on average) were required to produce a single LGE-CMR image using IR-FGRE. For MCLE, a segmented SSFP readout was used following an inversion recovery pulse, providing 20 cardiac phase-resolved images at different TIs [16]. The MCLE pulse sequence took about 13 heartbeats to acquire (one for the establishment of steady state, and the rest 12 for data acquisitions over an average breath-hold of 11 seconds). Typical CMR parameters for MCLE sequence were as follows: TR/TE 2.7/1.3 ms, rBW ±125 kHz, flip angle 30°, VPS 16, TDEL 500 ms, and NEX 1. The early recovery phase was also gated to diastole. The in-plane resolution was ~1.5×1.5 mm and the through-plane resolution was 8 mm for both LGE-CMR and SSFP pulse sequences.

**Figure 1:**
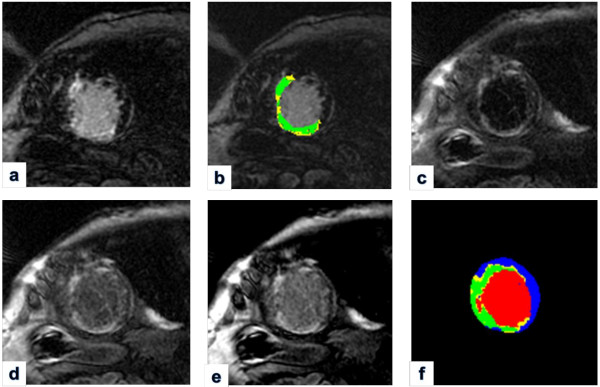
**IR-FGRE vs. MCLE peri-infarct GZ map. a**: IR-FGRE image; **b**: Peri-infarct GZ map corresponding to a; **c**-**e**: 3 MCLE images at varied TI; **f**: Peri-infarct GZ map from MCLE: yellow color indicating peri-infarct GZ, green color indicating core MI, red color indicating blood pool, and blue color indicating healthy myocardium.

LV functional parameters of LVEF, LV volumes at end-systolic (LVESV) and end-diastolic phase (LVEDV), stroke volumes (SV) and LV mass at end-diastolic phase were measured using CMR^42^ software (Circle Cardiovascular Imaging, Calgary, Canada). Infarct heterogeneity analysis of core MI and peri-infarct GZ in IR-FGRE used a full-width half-maximum method [14,19]. Epicardial and endocardial contours were manually drawn to segment pixels within the LV myocardium, and then a region of interest (ROI) was drawn in remote healthy myocardium. The mean (Mean_remote_), peak (Peak_remote_), and standard deviation (SD_remote_) of the signal intensities within the remote region were calculated. The cut-off values for the infarct core and peri-infarct GZ were applied using a FWHM approach with the following definitions: SI_core_ > 0.5 * Peak_infarct_ and Peak_remote_ < SI_gz_ < 0.5 * Peak_infarct_, where Peak_infarct_ is the peak signal intensity of all pixels in the infarct. Color mapping was used to demonstrate the core MI (green color) and peri-infarct GZ (yellow color, Figure 1). For MCLE, infarct heterogeneity analysis on core MI and peri-infarct GZ used a semi-automated data clustering algorithm and the details of this algorithm are elucidated in the reference [19]. For a succinct description, the signal intensity recovery based on MCLE images at diastolic phases of varied TIs (usually 6–8 images with minimal cardiac motion) was used to generate T1* and steady-state value maps. Using the fuzzy C-means algorithms on a scatter plot of T1* and steady state values, each voxel can be automatically characterized as infarct, healthy myocardium, or blood, with the fuzzy clustering algorithm identifying the probability of each voxel belonging to the above three clusters. The peri-infarct GZ was determined as the voxels of 25% to 75% probability belonging to infarct or healthy myocardium. A color map (Figure 1) was produced to represent the spatial distribution of blood pool (red color), core MI (green color), peri-infarct GZ (yellow color), and healthy myocardium (blue color). Similar to IR-FGRE analysis, isolated voxels within healthy myocardium classified as core MI or peri-infarct GZ (due to noise) were manually removed and re-labeled as healthy tissue. The extent of the core MI and peri-infarct GZ for each subject was expressed in grams of tissue and normalized to LV myocardium mass for both IR-FGRE and MCLE analysis.

The determination of peri-infarct GZ extent as well as PM-MI was blinded from the results of ICD events. The presence of PM involvement was considered if the following criteria were satisfied [18]: a) The increased signal intensity in PM was the same or similar to that of adjacent hyper-enhanced infarct segments on LGE-CMR images; b) The hyper-enhanced PM region was limited to the PM area defined by pre-contrast SSFP images. PM-MI score was determined to be 2, 1, or 0 if both of PM, only one PM, or no PM involvement was identified on LGE-CMR images using MCLE pulse sequence where there was a higher detection rate of PM-MI than in IR-FGRE data sets [18].

### ICD events and statistical analysis

All patients received a single or dual ICD implantation according to current clinical guidelines. All patients were followed in an ICD clinic at intervals of 3 months and more frequently (if device shocks were delivered) for 6–46 months with a median follow-up of 30 months. The ICD data for the relevant ventricular arrhythmic events was reviewed by two experienced electrophysiologists. The primary outcome measure was appropriate ICD therapy that was defined as shock for ventricular tachycardia (VT), ventricular fibrillation (VF), or any ventricular arrhythmic event identified as sustained VT or VF. Appropriate ICD therapy in the present study refers to an ICD event as that which was triggered for a single rhythm episode regardless of the total number of actual shocks that were needed for termination of tachycardia.

Categorical data are expressed as numbers (percentages) and compared with a Fisher exact test. Continuous variables are expressed as mean ± SD. The Student t-test was used for the statistical analysis of the proportion of peri-infarct GZ, core MI, total MI relative to LV myocardium mass, PM-MI scores, and LV functional parameters between groups with and without appropriate ICD therapy at follow-up. A p value <0.05 was defined as having a statistical significance.

## Results

The detailed demographic characteristics are presented in Table 1. All patients successfully completed the CMR study protocol and had ICD implantation according to existing guidelines and were followed at an ICD clinic for 6–46 months with a median of 30 months. Forty-eight percent of patients (12/25) had at least one appropriate ICD therapy for VF or sustained VT at follow-up. No cardiac death occurred during follow-up in the present study.

**Table 1:** Baseline patient characteristics and CMR LV functional parameters

**Patient characteristics**	**Total (n = 25)**	**With ICD therapy (N = 12)**	**Without ICD therapy (N = 13)**	**P value**
Age, years old	63.9 ± 10.0	63.7 ± 9.3	64.2 ± 10.9	0.91
Male	22 (88)	10 (83.3)	12 (92.3)	0.55
Primary prevention	15 (60)	6 (50)	9 (69.2)	0.44
NYHA functional class	1.48 ± 0.96	1.8 ± 1.0	1.2 ± 0.9	0.18
Anti-arrhythmic	5 (20)	2 (16.7)	3 (23.1)	0.57
Smoking	14 (56)	6 (50)	8 (61.5)	0.51
Hypertension	19 (76)	10 (83.3)	9 (69.2)	0.50
Diabetes	5 (20)	2 (16.7)	3 (23.1)	0.57
Hyperlipidemia	22 (88)	11 (91.7)	11 (84.6)	0.67
QRS duration (ms)	114.9 ± 29.0	120.7 ± 36.4	109.6 ± 20.2	0.35
Left bundle-branch block	5 (20)	3 (27.3)	2 (15.4)	0.50
**CMR LV function**				
LV EF (%)	26.2±10.4	22.1 ± 8.5	30.1 ± 10.9	0.054
LV ESV (ml)	179.6±81.1	203.5 ± 82.7	157.6 ± 76.2	0.16
LV EDV (ml)	236.6±82.2	256.2 ± 82.2	218.4 ± 81.2	0.26
LV SV (ml)	56.5±17.9	52.7 ± 18.9	60.1 ± 17.0	0.31
LVM (g)	106.1±29.9	110.9 ± 34.8	101.6 ± 25.2	0.45

Notes:

1. Continuous data are expressed as mean ± SD and categorical data as n (%).

2. *CMR* cardiovascular magnetic resonance, *ICD* Implantable cardioverter-defibrillator, *NYHA* New York Heart Association, *LV* left ventricular, *EF* ejection fraction, *ESV* end-systolic volume, *SV* stroke volume, *EDV* end-diastolic volume, *LVM* LV mass at end-diastolic phase.

3. P > 0.05 indicates no statistically significant difference between two groups.

The CMR image quality was acceptable in all 25 subjects for quantitative analysis of LV function and infarct heterogeneity such as core MI and peri-infarct GZ. The comparison on LV functional parameters from groups with and without appropriate ICD therapy was listed in Table 1 as well. There was no statistically significant difference observed from these two groups on functional parameters of LVEF, LVEDV, LVESV, SV and LVM although there was a trend toward poorer LV systolic function observed in patients with appropriate ICD therapy.

**Figure 2:**
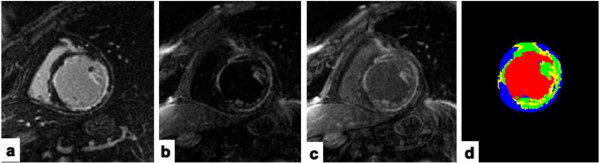
**A patient with ischemic heart disease demonstrating both PM involvement and a greater peri**-**infarct GZ of 18.5% determined by MCLE had ICD shocks for sustained VT at follow**-**up. a**: IR-FGRE image showing one PM involvement; **b**-**c**: MCLE images clearly demonstrating both PM involvement; **d**: Peri-infarct GZ map from MCLE: yellow color indicating peri-infarct GZ, green color indicating core MI, red color indicating blood pool, and blue color indicating healthy myocardium.

Infarct heterogeneity measurements including the total size of MI, core MI and peri-infarct GZ normalized to LV mass from groups with and without appropriate ICD therapy were listed on Table 2. There was no statistically significant difference observed in CMR infarct heterogeneity indices of core MI and total extent of MI relative to LV mass using either MCLE or IR-FGRE between these two groups, although the core MI and total extent of MI tended to be greater in subjects with appropriate ICD therapy from both MCLE and IR-FGRE measurements. However, the proportion of peri-infarct GZ relative to LV mass measured with MCLE as opposed to that measured with IR-FGRE demonstrated a statistical difference between subjects with and without appropriate ICD therapy for VF or sustained VT at follow up. PM-MI scores between groups with appropriate ICD therapy (1.67±0.49) and without (1.00±0.93) at follow-up demonstrated a statistically significant difference (p = 0.035). Figure 2 demonstrated such an example: a patient with ischemic heart disease and a greater amount of peri-infarct GZ relative to LV mass (18.5%) plus the presence of both PM involvements determined on MCLE images had ICD shocks for sustained VT at follow-up.

**Table 2:** CMR infarct heterogeneity measurements in subjects with and without ICD therapy

	**With ICD therapy (n = 12)**		**Without ICD therapy (n = 13)**		**P value**	
	**MCLE**	**IR**-**FGRE**	**MCLE**	**IR**-**FGRE**	**MCLE**	**IR**-**FGRE**
GZ/LVM (%)	14.8 ± 4.8	13.8 ± 5.1	11.2 ± 3.9	10.6 ± 5.1	0.046	0.14
Core MI/LVM (%)	25.8 ± 10.9	22.4 ± 9.9	19.6 ± 10.1	16.8 ± 10.9	0.15	0.19
Total MI/LVM (%)	40.6 ± 13.5	36.2 ± 14.8	30.9 ± 13.5	27.4 ± 15.8	0.08	0.16

Abbreviations: *CMR* cardiovascular magnetic resonance, *ICD* Implantable cardioverter-defibrillator, *MCLE* multi-contrast late enhancement, *IR*-*FGRE* inversion recovery fast gradient echo, *GZ* peri-infarct gray zone, *MI* myocardial infarction, *LVM* LV mass at end-diastolic phase.

## Discussion

ICD implantation has become a well-established therapy in patients with poor LV systolic function post myocardial infarction for primary prevention of sudden cardiac death [2,22]. However, rates of appropriate ICD therapy are generally low and searching for better markers to stratify patients at low or high risks for development of malignant VA is needed [5,23]. A recent study suggested that LGE-CMR infarct assessment is superior to LVEF for risk stratification as even in patients with low LVEF (<30%) a minimal or no scarring indicated a low-risk cohort similar in risk to those patients with higher LVEF (>30%) [24]. In the present study pre-selected for low LVEF, no significant differences were noted between CMR determined LV functional parameters from groups with and without ICD therapy, indicating that LVEF and LV EDV/ESV might not be highly sensitive markers in predicting appropriate ICD therapies.

Conventional LGE-CMR derived infarct tissue heterogeneity indices demonstrate the potential to accurately predict VA events in patients post myocardial infarction and might have a role in the triage of patients benefitting the most from ICD implantation [24-29]. This study further indicated that a new LGE-CMR (MCLE) derived peri-infarct GZ extent might be a more sensitive maker in the prediction of appropriate ICD therapy, compared to indices derived from conventional LGE-CMR. Peri-infarct GZ contains bundles of viable myocardium with a mixture of fibrotic scar tissue, indicating that re-entry routes for the slow electrical conduction might exist in the inhomogeneous infarct tissue, and whole mount histological techniques were attempted for validation [30,31]. On LGE-CMR images it appears as increased intermediate signal intensity (gray zone separating from the low signal intensity of healthy myocardium and high signal intensity of scar tissue) [19].

A higher sensitivity using MCLE in the identification of peri-infarct GZ could be illustrated inherently in its multi-contrast capability and the semi-automated data-clustering algorithm applied [16-19]. MCLE uses SSFP readout immediately after an inversion pulse, which permits visualization of infarction as an area of fast T1 recovery with the simultaneous nulling of blood pool and viable myocardium. The improved differentiation with multi-contrast among healthy myocardium, infarct and blood pool provides better identification of infarct heterogeneity. The use of semi-automated data-clustering algorithm may provide a more reproducible measurement of infarct heterogeneity, compared to other segmentation algorithms which frequently require manual contouring and arbitrary selection of ROIs in the remote region and core MI which can produce significant signal variation [32]. In this prospective study the extent of total MI and core MI did not achieve a statistical significance in the prediction of appropriate ICD therapy even using MCLE-based algorithms, probably due to the small sample size. However, in larger sample studies the extent of MI is a validated predictor for appropriate ICD shocks in patients with ischemic cardiomyopathy [24].

MCLE also provides better identification of PM involvement in patients with myocardial infarction [18]. This specific involvement is a recognized source of VA and surgical resection or ablation of scarred papillary muscle can eliminate ventricular arrhythmias [33,34]. This study demonstrated that PM-MI scores might have a specific role in the prediction of appropriate ICD therapy, although the exact mechanism warrants further exploration. PM-MI might indicate the existence of more inhomogeneous infarct tissue or a tendency of yielding a critical isthmus between scar tissues of LV free wall and papillary muscles. The efforts on identifying possible critical isthmuses have been reported using color overlay conventional LGE-CMR images [35,36]. These possible critical isthmuses might appear as distinguished channels of heterogeneous tissue corridors and were likely sites of slow conduction properties and the causes of VT events. As a large portion of PM are within LV cavity and attached to LV wall as a part of mitral apparatus [37], this morphology might provide the anatomic basis for more heterogeneous corridors existing between dense scar tissues of LV free wall and PM-MI, thus prompting the generation of malignant VA. In the routine analysis of infarct heterogeneity, the PM is often excluded as a portion of myocardium due to the time consuming effort needed to trace these structures or because of the poor delineation on conventional LGE-CMR using IR-FGRE techniques; thus the role of PM involvement in the prediction of adequate ICD therapy or worse outcome might be overlooked. The present study demonstrated that the improved detection of PM involvement in patients with prior MI using the MCLE pulse sequence might provide a new parameter of PM-MI scores in the risk stratification of adequate ICD implantation or for guiding the ablation of VT which originate from scarred papillary muscles.

There are several limitations to this study. First, this is a small sample study for testing the hypothesis that MCLE determined infarct heterogeneity indices might be more sensitive than conventional LGE-CMR in the prediction of ICD therapy in patients with ischemic heart disease. Due to the small sample size, multi-variable analysis was not feasible to determine which specific index was a better marker. In this study the higher ventricular arrhythmic episodes might be due to a selection bias. Patients were mixed population for secondary and primary prevention and had severe LV systolic dysfunction with an average infarct size of greater than 30% of LV mass indicating the existence of severe myocardial damage, which potentially contributed to the high ICD firing rates. Although this selection bias may affect the number of episodes, it should not affect their distribution between these two groups with or without appropriate ICD therapy in our opinion. The long period of follow-up (up to 4 years) might contribute to the higher ventricular arrhythmic episodes in this study as well.

## Conclusion

In conclusion, peri-infarct gray zone measurement using MCLE might be a more sensitive and reproducible parameter in predicting appropriate ICD therapy for malignant ventricular arrhythmias events, compared to using conventional LGE-CMR. Papillary muscle involvement scores might have a specific role in the prediction of appropriate ICD therapy as well although the exact mechanism needs further investigation.

## Abbreviations

ICD: Implantable cardioverter-defibrillator; CMR: Cardiovascular magnetic resonance; LGE: Late gadolinium enhancement; IHD: Ischemic heart disease; MCLE: Multi-contrast late enhancement; IR-FGRE: Inversion recovery fast gradient echo; GZ: Peri-infarct gray zone; SSFP: Steady-state free precession; FWHM: Full-width half-maximum; PM: Papillary muscle; MI: Myocardial infarction; NYHA: New York Heart Association; LV: Left ventricular; EF: Ejection fraction; ESV: End-systolic volume; SV: Stroke volume; EDV: End-diastolic volume; LVM: LV mass at end-diastolic phase; VA: Ventricular arrhythmia; VT: Ventricular tachycardia; VF: Ventricular fibrillation.

## Competing interests

Dr. Wright receives research funding from GE Healthcare.

## Authors’ contributions

YY: study design, collection, analysis and interpretation of data, and drafting of the manuscript. TZS, YL, GP, IR, MZ, JJG: collection, analysis and interpretation of data, critically review of the manuscript. KAC, AJD, EC, and GAW: study design, collection, analysis and interpretation of data, critically review of the manuscript. All authors have read and approved the final manuscript.

## References

[B1] Fishman GI Chugh SS Dimarco JP Albert CM Anderson ME Bonow RO Buxton AE Chen PS Estes M Jouven X Kwong R Lathrop DA Mascette AM Nerbonne JM O’Rourke B Page RL Roden DM Rosenbaum DS Sotoodehnia N Trayanova NA Zheng ZJ Sudden cardiac death prediction and prevention: report from a National Heart, Lung, and Blood Institute and Heart Rhythm Society Workshop Circulation 2010 122 2335 2348 10.1161/CIRCULATIONAHA.110.976092 21147730 PMC3016224

[B2] Myerburg RJ Implantable Implantable Cardioverter–Defibrillators after Myocardial Infarction N Engl J Med 2008 359 2245 2253 10.1056/NEJMra0803409 19020326

[B3] Kedia R Saeed M Implantable cardioverter-defibrillators: indications and unresolved issues Tex Heart Inst J 2012 39 335 341 22719141 PMC3368443

[B4] Epstein AE Dimarco JP Ellenbogen KA Estes NA III Freedman RA Gettes LS Gillinov AM Gregoratos G Hammill SC Hayes DL Hlatky MA Newby LK Page RL Schoenfeld MH Silka MJ Stevenson LW Sweeney MO **ACC**/**AHA**/**HRS 2008 guidelines for device**-**based therapy of cardiac rhythm abnormalities: ****executive summary** Heart Rhythm 2008 5 934 955 10.1016/j.hrthm.2008.04.015 18534377

[B5] Bardy GH Lee KL Mark DB Poole JE Packer DL Boineau R Domanski M Troutman C Anderson J Johnson G McNulty SE Clapp-Channing N Davidson-Ray LD Fraulo ES Fishbein DP Luceri RM Ip JH; Sudden Cardiac Death in Heart Failure Trial (SCD-HeFT) Investigators. **Amiodarone or an implantable cardioverter-defibrillator for congestive heart failure.** N Engl J Med 2005 352 225 237 10.1056/NEJMoa043399 15659722

[B6] Stecker EC Vickers C Waltz J Socoteanu C John BT Mariani R McAnulty JH Gunson K Jui J Chugh SS Population-based analysis of sudden cardiac death with and without left ventricular systolic dysfunction: two-year findings from the Oregon Sudden Unexpected Death Study J Am Coll Cardiol 2006 47 1161 1166 10.1016/j.jacc.2005.11.045 16545646

[B7] Kim HW Farzaneh-Far A Kim RJ Cardiovascular magnetic resonance in patients with myocardial infarction: current and emerging applications J Am Coll Cardiol 2009 55 1 16 10.1016/j.jacc.2009.06.059 20117357

[B8] Bello D Fieno DS Kim RJ Pereles FS Passman R Song G Kadish AH Goldberger JJ Infarct morphology identifies patients with substrate for sustained ventricular tachycardia J Am Coll Cardiol 2005 45 1104 1108 10.1016/j.jacc.2004.12.057 15808771

[B9] Strauss DG Wu KC **Imaging myocardial scar and arrhythmic risk prediction**--**a role for the electrocardiogram**? J Electrocardiol 2009 42 138 1 8 19185315 10.1016/j.jelectrocard.2008.12.010

[B10] Scott PA Morgan JM Carroll N Murday DC Roberts PR Peebles CR Harden SP Curzen NP The extent of left ventricular scar quantified by late gadolinium enhancement MRI is associated with spontaneous ventricular arrhythmias in patients with coronary artery disease and implantable cardioverter-defibrillators Circ Arrhythm Electrophysiol 2011 4 324 330 10.1161/CIRCEP.110.959544 21493964

[B11] Nazarian S Bluemke DA Lardo AC Zviman MM Watkins SP Dickfeld TL Meininger GR Roguin A Calkins H Tomaselli GF Weiss RG Berger RD Lima JA Halperin HR Magnetic resonance assessment of the substrate for inducible ventricular tachycardia in nonischemic cardiomyopathy Circulation 2005 112 2821 2825 10.1161/CIRCULATIONAHA.105.549659 16267255 PMC2943964

[B12] Iles L Pfluger H Lefkovits L Butler MJ Kistler PM Kaye DM Taylor AJ Myocardial fibrosis predicts appropriate device therapy in patients with implantable cardioverter-defibrillators for primary prevention of sudden cardiac death J Am Coll Cardiol 2011 57 821 828 10.1016/j.jacc.2010.06.062 21310318

[B13] Yan AT Shayne AJ Brown KA Gupta SN Chan CW Luu TM Di Carli MF Reynolds HG Stevenson WG Kwong RY Characterization of the peri-infarct zone by contrast-enhanced cardiac magnetic resonance imaging is a powerful predictor of post-myocardial infarction mortality Circulation 2006 114 32 39 10.1161/CIRCULATIONAHA.106.613414 16801462

[B14] Schmidt A Azevedo CF Cheng A Gupta SN Bluemke DA Foo TK Gerstenblith G Weiss RG Marbán E Tomaselli GF Lima JA Wu KC Infarct tissue heterogeneity by magnetic resonance imaging identifies enhanced cardiac arrhythmia susceptibility in patients with left ventricular dysfunction Circulation 2007 115 2006 2014 10.1161/CIRCULATIONAHA.106.653568 17389270 PMC2442229

[B15] Heidary S Patel H Chung J Yokota H Gupta SN Bennett MV Katikireddy C Nguyen P Pauly JM Terashima M McConnell MV Yang PC Quantitative tissue characterization of infarct core and border zone in patients with ischemic cardiomyopathy by magnetic resonance is associated with future cardiovascular events J Am Coll Cardiol 2010 55 2762 2768 10.1016/j.jacc.2010.01.052 20538171

[B16] Detsky JS Stainsby JA Vijayaraghavan R Graham JJ Dick AJ Wright GA Inversion-recovery-prepared SSFP for cardiac-phase-resolved delayed-enhancement MRI Magn Reson Med 2007 58 365 372 10.1002/mrm.21291 17654582

[B17] Connelly KA Detsky JS Graham JJ Paul G Vijayaragavan R Dick AJ Wright GA Multicontrast late gadolinium enhancement imaging enables viability and wall motion assessment in a single acquisition with reduced scan times J Magn Reson Imaging 2009 30 771 777 10.1002/jmri.21907 19787723

[B18] Yang Y Connelly K Graham JJ Detsky J Lee T Walcarius R Paul G Wright GA Dick AJ **Papillary** muscle involvement in myocardial infarction: initial results using multicontrast late-enhancement MRI J Magn Reson Imaging 2011 33 211 216 10.1002/jmri.22394 21182141

[B19] Detsky JS Paul G Dick AJ Wright GA Reproducible classification of infarct heterogeneity using fuzzy clustering on multicontrast delayed enhancement magnetic resonance images IEEE Trans Med Imaging 2009 28 1606 1614 19783498 10.1109/TMI.2009.2023515

[B20] Bogun F Desjardins B Crawford T Good E Jongnarangsin K Oral H Chugh A Pelosi F Morady F Post-infarction ventricular arrhythmias originating in papillary muscles J Am Coll Cardiol 2008 51 1794 1802 10.1016/j.jacc.2008.01.046 18452787

[B21] Doppalapudi H Yamada T McElderry HT Plumb VJ Epstein AE Kay GN Ventricular tachycardia originating from the posterior muscle in the left ventricle: a distinct clinical syndrome Circ Arrhythm Electrophysiol 2008 1 23 29 10.1161/CIRCEP.107.742940 19808390

[B22] Goldenberg I Gillespie J Moss AJ Hall WJ Klein H McNitt S Brown MW Cygankiewicz I Zareba W Executive Committee of the Multicenter Automatic Defibrillator Implantation Trial II Long-term benefit of primary prevention with an implantable cardioverter-defibrillator: an extended 8-year follow-up study of the Multicenter Automatic Defibrillator Implantation Trial II Circulation 2010 122 1265 1271 10.1161/CIRCULATIONAHA.110.940148 20837894

[B23] Zwanziger J Hall WJ Dick AW Zhao H Mushlin AI Hahn RM Wang H Andrews ML Mooney C Wang H Moss AJ The cost effectiveness of implantable cardioverter-defibrillators: results from the Multicenter Automatic Defibrillator Implantation Trial (MADIT)-II J Am Coll Cardiol 2006 47 2310 2318 10.1016/j.jacc.2006.03.032 16750701

[B24] Klem I Weinsaft JW Bahnson TD Hegland D Kim HW Hayes B Parker MA Judd RM Kim RJ Assessment of myocardial scarring improves risk stratification in patients evaluated for cardiac defibrillator implantation J Am Coll Cardiol 2012 60 408 20 10.1016/j.jacc.2012.02.070 22835669 PMC3424733

[B25] Bernhardt P Stiller S Kottmair E Binner L Spiess J Grossmann G Rasche V Walcher D Hombach V Myocardial scar extent evaluated by cardiac magnetic resonance imaging in ICD patients: relationship to spontaneous VT during long-term follow-up Int J Cardiovasc Imaging 2011 27 893 900 10.1007/s10554-010-9726-9 20957518

[B26] Roes SD Borleffs CJ van der Geest RJ Westenberg JJ Marsan NA Kaandorp TA Reiber JH Zeppenfeld K Lamb HJ de Roos A Schalij MJ Bax JJ Infarct tissue heterogeneity assessed with contrast-enhanced MRI predicts spontaneous ventricular arrhythmia in patients with ischemic cardiomyopathy and implantable cardioverter-defibrillator Circ Cardiovasc Imaging 2009 2 183 190 10.1161/CIRCIMAGING.108.826529 19808591

[B27] Wu KC Gerstenblith G Guallar E Marine JE Dalal D Cheng A Marbán E Lima JA Tomaselli GF Weiss RG Combined cardiac magnetic resonance imaging and C-reactive protein levels identify a cohort at low risk for defibrillator firings and death Circ Cardiovasc Imaging 2012 5 178 186 10.1161/CIRCIMAGING.111.968024 22267750 PMC3330427

[B28] Gao P Yee R Gula L Krahn AD Skanes A Leong-Sit P Klein GJ Stirrat J Fine N Pallaveshi L Wisenberg G Thompson TR Prato F Drangova M White JA Prediction of arrhythmic events in ischemic and dilated cardiomyopathy patients referred for implantable cardiac defibrillator: evaluation of multiple scar quantification measures for late gadolinium enhancement magnetic resonance imaging Circ Cardiovasc Imaging 2012 5 448 456 10.1161/CIRCIMAGING.111.971549 22572740

[B29] Dickfield T Pursuing the “Holy Grail” Circ Cardiovasc Imaging 2012 5 167 170 10.1161/CIRCIMAGING.112.972935 22438421

[B30] Yang Y Liu K Wang D Pop M Detsky J Dick AJ Yaffe MJ Wright GA Whole mount heart histology: a new gold standard for myocardial damage validation in experimental cardiac MRI studies? Proc Intl Soc Mag Reson Med 2010 18 3643

[B31] Pop M Ramanan V Yang Y Ghugre N Qiang B McVeigh ER Dick AJ Wright GA Comparision of scar morphology by 3D multi-contrast late enhancement MRI, 3D DW-MRI and histology in a pig model of chronic infarct [abstract] Proc Intl Soc Mag Reson Med 2010 18 3644

[B32] de Haan S Meijers TA Knaapen P Beek AM van Rossum AC Allaart CP Scar size and characteristics assessed by CMR predict ventricular arrhythmias in ischaemic cardiomyopathy: comparison of previously validated models Heart 2011 97 1951 1956 10.1136/heartjnl-2011-300060 21917670

[B33] Yokokawa M Good E Desjardins B Crawford T Jongnarangsin K Chugh A Pelosi F Jr Oral H Morady F Bogun F Predictors of successful catheter ablation of ventricular arrhythmias arising from the papillary muscles Heart Rhythm 2010 7 1654 1659 10.1016/j.hrthm.2010.07.013 20637311 PMC2970625

[B34] Kron IL DiMarco JP Nolan SP Lerman BB Resection of scarred papillary muscles improves outcome after surgery for ventricular tachycardia Ann Surg 1986 203 685 689 10.1097/00000658-198606000-00014 3718031 PMC1251206

[B35] Perez-David E Arenal A Rubio-Guivernau JL del Castillo R Atea L Arbelo E Caballero E Celorrio V Datino T Gonzalez-Torrecilla E Atienza F Ledesma-Carbayo MJ Bermejo J Medina A Fernández-Avilés F Noninvasive identification of ventricular tachycardia-related conducting channels using contrast-enhanced magnetic resonance imaging in patients with chronic myocardial infarction: comparison of signal intensity scar mapping and endocardial voltage mapping J Am Coll Cardiol 2011 57 184 194 10.1016/j.jacc.2010.07.043 21211689

[B36] Halperin HR Nazarian S Magnetic resonance identification of the ventricular tachycardia critical isthmus: finding the needle in the haystack J Am Coll Cardiol 2011 57 195 197 10.1016/j.jacc.2010.07.042 21211690 PMC3035155

[B37] Axel L Papillary muscles do not attach directly to the solid heart wall Circulation 2004 109 3145 3148 10.1161/01.CIR.0000134276.06719.F3 15197146

